# Toe Grip Strength Is Associated with Improving Gait Function in Patients with Subacute Stroke

**DOI:** 10.3390/brainsci14030215

**Published:** 2024-02-26

**Authors:** Jin-Whan Ryu, In-Su Hwang, Sol Jin, Soo-A Kim, Min-Su Kim

**Affiliations:** 1 Department of Rehabilitation Medicine, Soonchunhyang University Cheonan Hospital, Cheonan 31151, Republic of Korea; 129751@schmc.ac.kr (J.-W.R.); 135829@schmc.ac.kr (I.-S.H.); 138411@schmc.ac.kr (S.J.); sooapmr@schmc.ac.kr (S.-A.K.); 2 Department of Regenerative Medicine, College of Medicine, Soonchunhyang University, Cheonan 31151, Republic of Korea

**Keywords:** ambulation, foot, gait, recovery of function, rehabilitation, stroke, toe, walking

## Abstract

Toe grip strength has recently been suggested to play an essential role in maintaining balance and postural stability for ambulatory function in older populations. This study aimed to investigate its association with improving gait function three months after onset in patients with subacute stroke. This longitudinal cohort study included 98 first-ever stroke patients (67 ± 9 years, 56% female) within one month from the onset who could not ambulate independently. Functional outcome indicators, including toe grip strength, hand grip strength, knee extensor strength, Fugl-Meyer Assessment of Lower Extremity (FMA_LE), and the Postural Assessment Scale for Stroke (PASS), were assessed before and three months after the intervention. We analyzed the correlation between participants’ gait function using a 10-meter walk test time and various functional indicators. Then, multiple linear regression analysis was used to investigate whether toe grip strength was related to the improvement of gait function. Correlation analysis revealed a significant positive correlation between the 10MWT time and toe grip strength ratio (affected/unaffected side), with a moderate effect size (r = −0.61, *p* <0.001). Multiple regression analysis with covariates showed a significant relationship between 10MWT time and toe grip strength ratio (β = −0.113, *p* < 0.001), FMA_LE (β = −1.315, *p* = 0.004), PASS (β = −3.275, *p* <0.001), and age (β = −0.159, *p* = 0.004). In conclusion, toe grip strength was an essential factor associated with ambulatory function improvement in subacute stroke patients three months after onset. Additional toe grip muscle strengthening rehabilitation treatment can be expected to help improve the ambulatory function of subacute stroke patients in the future.

## 1. Introduction

Stroke is the leading cause of long-term gait impairment. As the survival rate of stroke patients has improved due to the development of acute stroke treatment, post-stroke gait disturbance prevalence is constantly increasing [1]. If stroke survivors are not able to ambulate independently, it is difficult for them to be discharged home, and they often stay in hospitals or rehabilitation facilities [2]. In addition, long-term gait impairment can further aggravate the disability status because it increases the incidence of cardiovascular disease in stroke patients [3]. Because this causes great costs and suffering to the patient’s family and society, improving gait function is one of the most important goals in stroke rehabilitation [4].

Many studies have been conducted to identify the factors involved in the recovery of gait function after stroke. Several longitudinal studies have reported that stroke survivors’ demographic characteristics, such as age and gender, and stroke-related features, such as stroke type, aspects, and location, may influence gait prognosis [5]. In addition, patients’ functional impairments, such as muscle strength, cognition, perception, vision, language, sitting balance, and activities of daily living, are also involved in gait function recovery [6]. Corticospinal tract integrity has been reported to influence the prognosis of post-stroke gait restoration in neuro-imaging studies [7]. It has recently been emphasized that improving balance and postural control function plays a vital role in independent ambulation after stroke [8].

While various systems are involved in the static and dynamic balance capability of the musculoskeletal, neuromuscular, sensory/perceptual, and cognitive systems, toe strength has recently been suggested to play an essential role in maintaining balance during ambulation [9]. Utani et al. [10] reported that toe grip strength was significantly correlated with dynamic balance and functional mobility in a study that examined the association between toe grasping strength and functional mobility levels in adults aged 60 years and older. In addition, it was announced that an age-related decline in toe grip muscle strength was significantly associated with poor walking speed and performance [11,12]. Another study reported that weakness in toe flexor muscles increased the frequency of wheelchair use in older adults [13].

Several studies investigated the effect of toe grip-strengthening exercises. Kojima et al. [14] conducted a randomized controlled study in which home-based toe-grasping exercises were applied to elderly patients for three months, and their effects were analyzed. As a result, it was reported that standing balance improved significantly [14]. Another randomized controlled study in which strengthening exercises were performed using an insole with a toe grip bar in patients with Parkinson’s disease reported significant improvements in balance and gait function [15].

However, few studies have examined whether hemiplegic toe grip strength is related to gait function in stroke patients. Therefore, this study aimed to determine the degree of deterioration of toe grip strength in subacute stroke patients and investigate whether it is associated with improving gait function three months after onset. The study of the association between toe grip strength and the recovery of gait function could be the basis for developing additional stroke rehabilitation techniques to improve gait function in the future.

## 2. Materials and Methods

### 2.1. Participants

This longitudinal cohort study included first-ever stroke patients within one month from the onset who could not ambulate independently. The patients’ ages ranged from 20 to 79 years, and ischemic or hemorrhagic stroke were diagnosed by neurologists and neurosurgeons based on brain magnetic resonance imaging (MRI) or computed tomography (CT) scan. Among the participants, those who could not perform the 10 m walk test (10MWT) and those with gait disturbance before stroke were excluded. Patients with severe cognitive impairment and foot deformities who could not perform toe grip strength tests were also excluded. All caregivers and participants provided written informed consent and were free to stop the study at any time.

### 2.2. Toe Strength Measure

**Figure 1:**
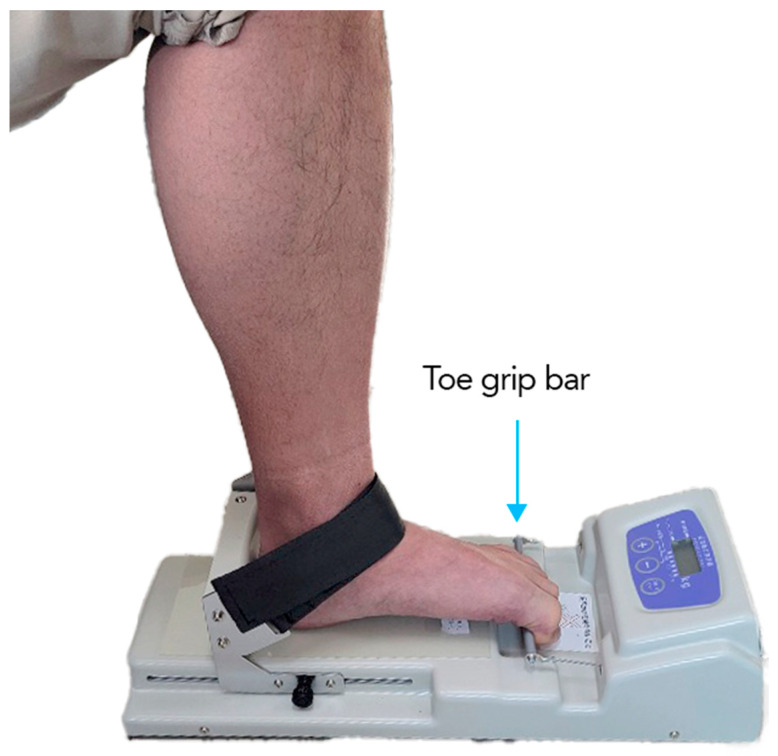
Measurement of toe strength. The patient’s toe strength was measured in a sitting position. The ankle was placed in a neutral position and secured with a strap. The first proximal interphalangeal joint was positioned on the grip bar, and the heel stopper was adjusted to fit the heel of each participant. The patient then grabbed the bar of the measuring instrument with maximum force for about 2 s using his toes. All patients were measured twice, and the mean value was used as an indicator of the patient’s toe strength.

Toe strength was assessed using the T.K.K.3362 toe-grip dynamometer (Takei Scientific Instruments, Niigata, Japan). This device has proven reliable and valid in normal adults aged 20–79 years [16]. Patients were evaluated in wheelchairs or chairs two weeks after onset. The hip joint and knee were bent about 90°, the ankle was placed in a neutral position, and the foot was fixed with a strap (Figure 1). The first proximal phalanx was placed on the grip bar, and the heel stopper was adjusted to the heel of each participant. Participants practiced using their normal toes with less than maximum effort before the actual measurement was made, and then the test was performed. The strength of the toes on the unaffected side was measured first, and then the toe strength on the affected side was measured. Two measurements were taken for each toe, and the mean value was obtained. In addition, the ratio of the affected side to the toe strength of the unaffected side was calculated (affected side strength/unaffected side strength, %).

### 2.3. Other Behavioral Measurements

All behavioral indicators, including toe grip strength, were assessed before the beginning of rehabilitation and after three months. Gait function was evaluated using 10MWT, a tool used to assess the gait function of stroke patients and the time spent while the patient is walking 10 m [17]. Participants could wear AFOs and use walking aids, including single canes, quad canes, and walkers. Patients were instructed to walk at an average, comfortable pace, and the physical therapist did not assist them while the test was performed [18]. The test was performed twice, and the shortest walking time was recorded.

Hand grip strength was measured using a digital hand dynamometer (EH101, Zhongshan Camry Electronic Co., Ltd., Zhongshan, China). The patients were made to sit on a straight-backed armless chair with their feet flat on the floor, elbow flexed at 90°, and the dynamometer was held by the testing hand in a neutral grip [19]. Measurements were taken twice, alternating between affected and unaffected hands, and the mean value of each was obtained. Isometric knee extension strength was assessed with a microFET IITM (Hoggan Health Industries, Draper, UT, USA). During the measurement, the subjects were in a supine position, with their arms lightly positioned on their chest. The measurements consisted of two trials of 3-s maximal isometric contractions of the affected and unaffected knee extensors [20].

Functional outcome indicators included the Fugl-Meyer Assessment of Lower Extremity (FMA_LE) and the Postural Assessment Scale for Stroke (PASS). The FMA-LE is a widely used scale for assessing motor function after stroke [21]. The evaluation items comprise an assessment of reflex activity, voluntary movements within and outside of synergies, the ability to perform isolated movement, and coordination, and the highest score is 34 points [22]. The Postural Assessment Scale for Stroke (PASS) is an outcome measure specifically designed to assess and monitor postural control after stroke [23]. It involves 12 items, the score for which can vary from 0 to 3, with 0 being the lowest level of functionality and 3 the highest; the maximum total score is 36 [24].

Clinical and demographic information, including age, sex, stroke type, lesion side, the period after stroke onset, comorbidities, National Institutes of Health Stroke Scale (NIHSS) score at the onset, and the Korean version of Mini-Mental State Exam (K-MMSE), were collected.

Participants received one month of comprehensive inpatient rehabilitation therapy, including physical and occupational therapy, at the tertiary hospital. After discharge, the patients underwent outpatient rehabilitation therapy five times a week for two months.

### 2.4. Statistics

To calculate the sample size for correlation, we used a two-tailed test with the following parameters: effect size (r) = 0.3, α error = 0.05, and power = 0.8, indicating the need for 82 participants. The sample size required for multiple regression analysis was calculated as effect size (f^2^) = 0.15, α error = 0.05, and power = 0.8, and 6 independent variables were required. Therefore, a total of 98 participants were included in this study. G*Power 3.1.9.7 was used for sample size calculation.

The Kolmogorov–Smirnov test was used to verify the normal distribution of data. A paired *t*-test or Wilcoxon signed-rank test was used to identify whether there were any significant changes in walking speed and functional indicators at baseline and after three months. Pearson correlation analyses were performed to investigate whether functional indicators with significant changes in the paired *t*-test after three months correlated with improvements in walking speed. Multiple regression analysis was performed with the variable with the significant correlation found as the independent variable and the change in walking speed as the dependent variable. Age, which may act as a confounding variable, was also included as a covariate. Analysis of variance (ANOVA) was used to confirm the validity of the regression equation, and the fit of the multiple regression equation was determined using the R^2^ value. Based on the coefficient of dispersion expansion (VIF), the multicollinearity of the multiple regression formula was determined. A *p*-value of below 0.05 was defined as statistically significant, and all statistical analyses were performed using SPSS Statistics v.29.0 (IBM SPSS Statistics for Windows, IBM Corp., Armonk, NY, USA).

## 3. Results

### 3.1. Baseline Demographic and Clinical Characteristics of the Participants

A total of 102 patients were enrolled in this study. Of these, four patients were excluded due to severe ankle or finger spasticity (modified Ashworth Scale ≥ 2) to guarantee a precise measure of the grip strength and hand grip strength. Therefore, 98 patients (67 ± 9 years, 56% female) were included in the final analysis. The participants’ characteristics are shown in Table 1.

### 3.2. Change in Functional Indicators after Three Months

After three months from the baseline, the 10MWT time decreased significantly from an average of 30 s to 13 s. The toe grip strength of the affected side increased significantly from 5.5 kg to 6.8 kg, and the toe grip strength ratio increased significantly from 60% to 74% on average. There was no significant difference in the grip strength on the unaffected side before and after three months. The hand grip strength of the affected side increased from 2.8 kg to 3.1 kg, but it was not statistically significant. The knee extensor strength on the affected side tended to increase from 54 N to 61 N but was also not statistically significant. The FMA_LE increased significantly from an average of 18 to 23 points, and PASS improved significantly from 13 to 19 points. Table 2 summarizes the changes in the 10MWT and functional indicators.

### 3.3. Correlation Analysis between the Walking Speed and the Functional Factors

Table 3 presents the results of the correlation analyses. The change in the time it takes to perform 10MWT over three months was found to be significantly negatively correlated with the improvement in the toe grip strength ratio (r = −0.61, *p* < 0.001). In addition, the change in FMA_LE (r = −0.58, *p* = 0.004) and PASS (r = −0.67, *p* < 0.001) was significantly negatively correlated with the change over three months of 10MWT. Age was significantly negatively correlated with 10MWT (r = −0.49, *p* = 0.004).

### 3.4. Association between Toe Grip Strength and Gait Function Improvement

Table 4 shows the results of the regression analyses. Multiple linear regression analysis was performed using significant indicators for the correlation analysis. The improvement in walking speed at three months was significantly related to the enhancement in the toe grip strength ratio (B = −0.099, *p* ≤ 0.001). In addition, the increase in walking speed was related to the improvement in FMA_LE (B = −0.083, *p* = 0.004) and PASS (B = −0.166, *p* ≤ 0.001). Age was also a significant factor influencing the degree of improvement in walking speed (B = −0.208, *p* = 0.004). The results of the ANOVA for this model were significant (*p* < 0.001), with an R^2^ value of 0.796. No variables with VIF > 5 were identified.

## 4. Discussion

We investigated whether toe grip strength is a factor involved in improving walking function in people with subacute stroke. The analysis, which included several demographic, clinical, and functional indicators known to affect gait function, showed that toe grip strength was associated with improving gait function at three months in patients with subacute stroke. In addition, age, lower limb strength, and postural control function, along with toe strength, were found to be factors involved in the positive recovery of gait function.

Multiple regression analysis showed a significant association between gait function improvement and the toe grip strength ratio. Three months after the onset of the stroke, there was no change in toe strength on the unaffected side, but there was an increase in toe strength on the affected side, so the toe grip strength ratio was increased. The mechanism by which toe grip strength contributes to gait can be speculated from previous studies. Toe strength can contribute to an exercise that accelerates the center of pressure (COP) in the terminal stance phase [25]. In this phase, about 20~30% of the body weight is applied to the toes, and the toes perform essential movements [26]. Older people, in particular, have been known to experience increased pressure on all toes instead of just the big toe when walking [27]. Past studies have shown that while walking, the COP shifts from the heel toward the big toe and that increased toe grip strength can contribute more to the forward movement of the COP [28]. We speculated that improvement of toe grip strength in stroke patients contributed to enhancing gait function by helping the COP movement during ambulation.

The study’s results showed that an increase in the toe strength ratio was significantly associated with improved ambulatory function rather than an improvement in toe strength itself. Unlike participants in previous studies where the association between toe strength and gait function was examined, stroke patients had a significant difference in bilateral toe strength due to hemiparesis. After three months, there was no change in toe strength on the unaffected side, but there was a gradual increase in toe strength on the affected side. The results of this study implicated that symmetrical muscle strength in both toes is important for maintaining balance in stroke patients, and that balance function is strongly related to the improvement in gait function. Previous studies that have suggested the importance of balance function in improving gait function have focused on the truncal function required to maintain sitting balance [29]. The symmetry of the trunk and its body-balancing function have been reported to have a significant impact on independent gait after stroke [6,30]. In the upright position, the timely and adequate generation of a plantar flexor torque by the toe flexors prevents the anterior displacement of the center of mass beyond the base support [31]. As a result, this study showed that toe flexor muscle strengthening rehabilitation on the affected side rather than the unaffected side is necessary to improve ambulatory function in stroke patients.

In addition to toe strength, age, motor function of the lower leg, and postural control function were also analyzed as potential predisposing factors that can predict gait function improvements three months after onset in the early stages of stroke onset. In previous studies, these have been identified to be predisposing factors that can predict independent gait, and our findings showed concordant results [5,32]. However, cognitive function and activities of daily living level, which were cited as predictors in previous studies, were not significant indicators in this study. It is speculated that these differences are due to the different characteristics of patient populations across various studies. Participants in this study had a large proportion of infratentorial cerebral infarcts encroaching on the posterior circulation system. In particular, East Asians, such as Koreans and Chinese, are more likely to suffer from posterior circulation ischemic stroke than Caucasians [33,34]. Patients with posterior circulation ischemic stroke are more likely to complain of vertigo and ataxia than motor weakness, and gait disturbance often occurs due to these symptoms [35]. In future studies, it would be necessary to develop a customized gait function prediction tool tailored to the characteristics of the patient group by considering the patients’ demographic, clinical, functional, and brain imaging characteristics.

## 5. Conclusions

In conclusion, toe grip strength was an essential factor associated with ambulatory function improvement in subacute stroke patients three months after onset. Furthermore, increased symmetry of toe grip strength may contribute to improved ambulatory function in stroke patients by improving balance and postural control. In the future, additional toe grip muscle strengthening rehabilitation treatment is expected to help improve the ambulatory function of subacute stroke patients.

## Figures and Tables

**Table 1:** Baseline demographic and clinical characteristics of the participants.

Factor		Value (*n* = 98)
Age	(years)	67.5 ± 9.5
Sex	Male	43
	Female	55
Stroke type	Ischemic	68
	Hemorrhagic	30
Lesion side	Right	45
	Left	53
Period after stroke onset	(days)	14.3 ± 5.2
NIHSS score	(onset)	9.1 ± 4.1
Comorbidity	Hypertension	91
	Diabetes	38
	Hyperlipidemia	62
K-MMSE	(at the beginning of the study)	20.1 ± 6.9
AFO	Yes	60
	No	38
Walking-assistance device	No device	7
	Single cane	39
	Quad cane	44
	Walker	8
MAS of ankle and fingers	0	84
	1	14
	1+	0
	2	0
	3	0
	4	0

Values are presented as a number (%) or mean ± standard deviation. NIHSS, National Institutes of Health Stroke Scale; K-MMSE, Korean version of Mini-Mental State Exam; AFO, Ankle-Foot Orthosis; MAS, Modified Ashworth Scale.

**Table 2:** Change in functional indicators after three months.

Factors	Baseline	After 3 Months	*p*-Value ^†^
10 m walk test (s)	30 ± 9	13 ± 3	<0.001 *
Toe grip strength, affected (kg)	5.5 ± 3.0	6.8 ± 3.4	0.032 *
Toe grip strength, unaffected (kg)	9.1 ± 3.2	9.2 ± 3.2	0.915
Toe grip strength ratio (%)	60 ± 8	74 ± 9	<0.001 *
Hand grip strength, affected (kg)	2.8 ± 1.5	3.1 ± 1.2	0.101
Hand grip strength, unaffected (kg)	6.3 ± 2.7	6.1 ± 2.6	0.496
Hand grip strength ratio (%)	44 ± 12	47 ± 13	0.091
Knee extensor strength, affected (N)	42 ± 16	46 ± 19	0.081
Knee extensor strength, unaffected (N)	91 ± 28	90 ± 32	0.517
Knee extensor strength ratio (%)	46 ± 19	51 ± 24	0.024 *
FMA_LE	18 ± 4	23 ± 4	0.004 *
PASS	13 ± 1	19 ± 4	<0.001 *

Values are presented as a number (%) or mean ± standard deviation. FMA_LE, Fugl-Meyer Assessment of Lower Extremity; PASS, Postural Assessment Scale for Stroke. ^†^: Paired *t*-test. *: *p* < 0.05.

**Table 3:** Correlation analysis between the walking speed and the functional factors associated with post-stroke gait improvement.

Pearson Correlation Coefficient (r)	Δ10MWT	ΔToe Grip Strength, Affected	ΔToe Grip Strength Ratio	Δknee Extensor Strength Ratio	ΔFMA_LE	ΔPASS	Age
ΔToe grip strength, affected	−0.43	-	-	-	-	-	-
ΔToe grip strength ratio	−0.61 *	0.86 *	-	-	-	-	-
ΔKnee extensor strength ratio	−0.43	0.29	0.51	-	-	-	-
ΔFMA_LE	−0.58 *	0.40 *	0.55 *	0.49 *	-	-	-
ΔPASS	−0.67 *	0.58	0.62 *	0.39	0.33	-	-
Age	−0.49 *	−0.46 *	−0.40	−0.38	−0.34 *	−0.44	-

10MWT, 10 m Walk Test; FMA_LE, Fugl-Meyer Assessment of Lower Extremity; PASS, Postural Assessment Scale for Stroke. *: *p* < 0.05.

**Table 4:** Functional factors associated with the post-stroke gait improvement over 3 months.

Independent Variable	Unstandardized Coefficient (B)	Standardized Coefficient (β)	t	*p*-Value	VIF
ΔToe grip strength ratio	−0.099	−0.113	−2.093	<0.001 *	1.492
ΔFMA_LE	−0.083	−0.142	−1.315	0.004 *	1.601
ΔPASS	−0.166	−0.213	−3.275	<0.001 *	1.883
Age	−0.208	−0.252	−0.159	0.004 *	1.922

FMA_LE, Fugl-Meyer Assessment of Lower Extremity; PASS, Postural Assessment Scale for Stroke. Multiple regression analysis, ANOVA < 0.001, adjusted R^2^ = 0.796. * *p* < 0.05.

## Data Availability

The datasets analyzed during the current study are available from the corresponding author on reasonable request. The data are not publicly available due to privacy or ethical restrictions.
